# Chimeric Antigen Receptor T-Cells in B-Acute Lymphoblastic Leukemia: State of the Art and Future Directions

**DOI:** 10.3389/fonc.2020.01594

**Published:** 2020-08-26

**Authors:** Uri Greenbaum, Kris Michael Mahadeo, Partow Kebriaei, Elizabeth J. Shpall, Neeraj Y. Saini

**Affiliations:** Department of Stem Cell Transplantation and Cellular Therapy, The University of Texas MD Anderson Cancer Center, Houston, TX, United States

**Keywords:** chimeric antigen receptor, acute lymphoblastic leukemia, CAR-T therapy, B-ALL, relapse after CAR-T therapy, allogeneic transplant after CAR-T therapy

## Abstract

Use of adoptive T-cell therapy modified with chimeric antigen receptor (CAR-T) has revolutionized treatment of patients with relapsed/refractory (r/r) B-cell acute lymphoblastic leukemia (B-ALL). CAR-T cells directed against CD19 antigen have produced response rates as high as 90% in clinical trials for r/r B-ALL. Despite high rates of complete remissions, the durability of responses has been sub-optimal with frequent relapses, especially in adult B-ALL population. Systemic toxicities from CAR-T therapy and standardization of toxicities grading and management is another major hurdle in the development of CAR-T field. In this review, we discuss the latest evidence of CAR-T therapy in B-ALL, potential mechanisms of relapse and barriers to CAR-T cell therapy in B-ALL. We also debate the role of allogeneic hematopoietic stem cell transplant (allo-HCT) post CAR-T therapy.

## Introduction

Use of adoptively transferred T cells modified with chimeric antigen receptors (CAR-T) has heralded a new era in the treatment of hematological malignancies, with unparalleled survival outcomes seen in patients with relapsed or refractory (r/r) disease (1–4). The idea of adoptive immunotherapy using T cells to attack cancer was developed in the early 1990s, and the first CAR was conceived by Eshhar et al. (5). CARs are synthetic receptors which include an extracellular target binding domain combined with a signaling domain, typically CD3zeta, plus costimulatory domains (single or in combination) from multiple genes such as CD28, 4-1BBL, and OX40. Over the past decade, the field of adoptive T therapy has progressed at an impressive pace, both in clinical field and in the development of innovative CAR-based platforms to improve the safety and efficacy of these therapies. In August 2017, a major milestone in the treatment of r/r B-cell acute lymphoblastic leukemia (B-ALL) was achieved with FDA approval of the first gene therapy (Novartis) (6), a CD19-targeted CAR-T cell–based product, tisagenlecleucel (CTL019) for B-ALL in children and young adults up to 25 years of age. Shortly thereafter, Gilead Pharma's CD19 CAR-T product, axicabtagene ciloleucel, obtained FDA approval for adult patients with r/r diffuse large B-cell lymphoma (7).

B-ALL is the most common type of acute leukemia in children in the United States, with an annual incidence of ~3,000 cases per year (8). In children, OS exceeds 85% (9); however, in adults, OS has been poor, ranging up to 50–60% (10, 11). Frontline induction chemotherapy regimens in ALL induces high rates of complete remission (CR), of up to 90%, but ~40–50% of adult ALL patients will eventually relapse. The outcomes in r/r ALL are even more dismal, with CR rates of only 30–40% with first salvage and only up to 10% with second salvage (12–14). With promising results from multiple trials in r/r ALL, CAR-T therapy has been added as a vital part of the therapeutic armamentarium for this disease.

The development of novel agents such as monoclonal antibodies (anti-CD20), anti-CD19 bispecific T-cell engager (blinatumomab), and anti-CD22 antibody-drug conjugate (inotuzumab ozogamicin) has provided excellent results in both upfront and r/r ALL and continues to change the treatment paradigm for ALL (15–18). However, the durability of responses achieved from these novel agents used as single-agent treatments in r/r ALL is dismal (15, 16) and would probably be better if these agents were used in combination. Although there is no definitive randomized trial or retrospective studies comparing these novel agents with CAR-T therapy, recent data from multiple studies have positioned CAR-T therapy with a significant edge over these novel agents due to better CR rates and better efficacy in r/r ALL. However, combinations of these novel agents with CAR-T therapy, either sequentially or as a maintenance strategy, have the potential to further improve survival outcomes. The review of literature pertaining to novel agents in ALL is beyond the scope of this review. In this review, we focus on current evidence in the literature for the efficacy and safety of CAR-T therapy in ALL and discuss the role of allogeneic hematopoietic stem cell transplantation (allo-HCT) in ALL patients who receive CAR-T therapy.

## CD19 CAR-T Clinical Trials in B-ALL

CD19 is uniformly expressed on all B-ALL cells and remains the most widely used target for CAR-T adoptive cell therapy. CD19 is also expressed on normal B cells, and despite the on-target, off-tumor toxicity of B-cell aplasia with hypogammaglobinemia, patients do well in the short term, and those with recurrent infections can receive therapy with intravenous immunoglobulin (IVIG) supplementation. Initial studies using CAR-T cells for B-cell malignancies showed promising preliminary results in indolent lymphomas and chronic lymphocytic leukemia (19–21). A few years later, two complete remissions in pediatric B-ALL were described (22). In a pilot clinical trial led by the Children's Hospital of Philadelphia (CHOP) and the University of Pennsylvania and published in 2014, 25 children and 5 young adults with r/r B-ALL were treated with CD19 CAR (1). These patients had been heavily pretreated, and in 60%, disease relapsed after allo-HCT. CR by morphology was achieved in 27 patients (90%), 73% of whom obtained minimal residual disease (MRD)-negative CR as assessed by flow cytometry.

Since then, multiple early-phase trials and later, larger multicenter trials have established the safety and efficacy of CD19 CAR-T therapy (3, 21, 23–26). Larger clinical trials led to the first commercially available product, tisagenlecleucel, which was approved by the FDA in 2017. In the pivotal ELIANA trial (3), which led to this approval, 75 children and young adults were treated with CD19 CAR-T cells. The overall remission rate within 3 months was 81% (61/75), with all patients who had a response to treatment found to be negative for MRD by flow cytometry. The 6-month event-free survival (EFS) and OS rates were 73 and 90%, respectively. The 1-year EFS and OS rates were 50 and 76%, respectively.

Table 1 lists major trials of CD19 CAR-T therapy in patients with r/r B-ALL. These trials varied widely by CAR vector constructs, eligibility criteria, patient population, and dosing schemes; however, similar unprecedented CR rates that were achieved in almost all trials imparted credibility to CAR-T therapy in general. While tisagenlecleucel contains the 4-1BB costimulatory domain, Memorial Sloan Kettering Cancer Center conducted a trial using a CAR construct with a CD28 costimulatory domain, enrolling 53 adult patients with relapsed B-ALL (23). Complete remission was observed in 83% of the patient population. Among patients who were assessed for MRD by flow cytometry, 67% had an MRD-negative CR. The median EFS and OS durations among the 53 treated patients were 6.1 and 12.9 months, respectively.

**Table 1:** Rates of response, CRS, and ICANS in major CD19 CAR T-cell clinical trials.

**References**	**Age**	**Product**	**Co-stim**	**No**	CRS^*^	sCRS^*^	ICANS^*^	sICANS^*^	**CR**	**Prolonged response**
Lee et al. (26)	Ped+YA	CD28 containing CAR	CD-28	51	N/A	7 (14%)	N/A	5 (10%)	61%	Median LFS^$^, 18 mo
Gardner et al. (27)	Ped+YA	1:1 CD4:CD8	CD28+ 41BB	43	40 (90%)	10 (23%)	21 (49%)	9 (21%)	93%	12-mo EFS, 50.8%
Maude et al. (3)	Ped+YA	Tisagenlecleucel	41BB	75	58 (77%)	35 (44%)	30 (40%)	10 (13%)	81%	50%
Pasquini et al. (28)^#^	Ped+YA	Tisagenlecleucel	41BB	144	85 (59%)	19 (13%)	42 (29%)	12 (8%)	89%	6-mo LFS, 66%
Turtle et al. (25)	Adults	1:1 CD4:CD8	CD-28 + 41BB	30	25 (83%)	7 (23%)	15 (50%)	15 (50%)	90%	43% at 6 mo
Hay et al. (29)	Adults	1:1 CD4:CD8	CD28 + 41BB	47^@^	35 (70%)	4 (12%)	11 (40%)	14 (21%)	NA	NA
Park et al. (23)	Adults	MSK CAR-T	CD28	53	45 (85%)	14 (26%)	24 (46%)	22 (42%)	83%	Median EFS, 6.1 mo
Shah et al. (30)	Adults	KTE-X19	CD28	45	N/A	13 (29%)	N/A	17 (38%)	73%	Median EFS, 15 mo

* *Different trials used different toxicity grading systems*.

$ *Among responders*.

# *Post-marketing CIBMTR data*.

@ *47 ALL patients of 133 patients with CD19 malignancies*.

*ALL, acute lymphoblastic leukemia; CRS, cytokine release syndrome; ICANS, immune effector cell–associated neurotoxicity syndrome; Ped+YA, pediatrics and young adults; Co-stim, costimulatory domain; CR, complete response; CIBMTR, Center for International Blood and Marrow Transplant Research*.

Axicabtagene ciloleucel (KTE-X19), another CD19 CAR-T product with a CD28 costimulatory domain, has been designated a US FDA breakthrough therapy for non-Hodgkin lymphoma. In a phase 1 trial, 45 adult patients with r/r B-ALL received KTE-X19 at one of three different doses (2 × 10^6^ cells/kg [*n* = 6], 1 × 10^6^ cells/kg [*n* = 23], or 0.5 × 10^6^ cells/kg [*n* = 16]) (31). Of 41 patients who were evaluable for efficacy, 68% achieved CR or CR with incomplete hematological recovery (CRi), and 100% of responders had undetectable MRD. At a dose of 1 × 10^6^ cells/kg, 16 of 19 patients with ≥ 2 months of followup (84%) achieved CR or CRi.

These trials, despite variation in CAR constructs and manufacturing, have consistently shown that CD19 CAR-T therapy induces high CR rates in high-risk, heavily pretreated patients with r/r B-ALL. Real-world experience from post-marketing registry data from the Center for International Blood and Marrow Transplant Research (CIBMTR) demonstrate similar results to those of preceding clinical trials, with 89% of 96 patients achieving a CR, and in patients whose MRD data were available (82% of patients), all were MRD-negative (28). This cohort included children and young adults and showed a 66% leukemia-free survival rate and 89% OS at 6 months. Further, various populations with B-ALL with historically poorer outcomes, such as those with Ph+ disease, patients whose disease relapsed after allo-HCT, and even patients with extra medullary disease and central nervous system (CNS) involvement, have responded well to CAR-T therapy. In another study of 12 patients with CNS ALL involvement before CAR-T therapy, no patients experienced CNS relapse (32).

Aside from the unique systemic toxicities associated with CAR-T therapy, the major challenge to CAR-T therapy has been difficulty in obtaining durable responses, especially in the adult B-ALL population. Despite initial impressive deep responses obtained with this therapy, more than half of the adult B-ALL patients experience relapse (22, 23, 26, 33–37) if not bridged to allo-HCT. Moreover, we are currently unable to accurately predict which patients will achieve long-term remission and/or persistence of *in vivo* CAR-T. As CAR-T and gene therapy fields continue to evolve, we will likely see more effective products aimed at improving the potency, safety, and persistence of CAR-T therapy.

## Toxicities Associated With CAR-T Therapy

The toxicities associated with CAR-T therapy range broadly, from on-target, off-tumor effects such as B-cell aplasia/hypogammaglobulinemia to immune mediated effects such as cytokine release syndrome (CRS) and immune effector cell–associated neurotoxicity syndrome (ICANS). CRS is characterized by signs and symptoms ranging from fever to widespread systemic life-threatening sequelae such as hypotension, hypoxia, and multiorgan dysfunction due to an immune-mediated cytokine storm caused by the expansion of the CAR-T cells (29). The severity of CRS almost always correlates with elevation of cytokines and chemokines such as IL-6, 1L-8, IL-10, interferon γ, and monocyte chemoattractant protein 1 (MCP-1) (29). The incidence of CRS in ALL and NHL patients treated with tisagenlecleucel was 77% (3) and 57% (2), respectively. The incidence of severe CRS in ALL and NHL patients was about 46 and 18%, respectively. In contrast, the incidence of severe CRS with axicabtagene ciloleucel in ALL and NHL patients was 13 and 29%, respectively.

ICANS clinically manifests with the deterioration of neurological function starting from word-finding difficulty with stuttering, writing impairment, and decreased concentration and progressing to more severe cases with a depressed level of consciousness, convulsive or non-convulsive seizures, and at times raised intracranial pressure/cerebral edema (38). The pathophysiology of ICANS is still not completely understood, and the mechanism is believed to be related to endothelial activation and blood-brain barrier disruption. The severity of ICANS correlates with elevated cytokine levels as well as with the rate of CAR-T expansion (39). The incidence of neurotoxicity in ALL and NHL patients treated with tisagenlecleucel is about 40% (3) and 39% (2), respectively. Severe neurotoxicity is seen in about 13 and 11% of ALL and NHL patients respectively. In contrast, the incidence of severe neurotoxicity with axicabtagene ciloleucel in ALL and NHL patients is ~38 and 28%, respectively.

ICANS may occur concurrently with CRS and/or without associated CRS. Host and tumor factors such as higher tumor burden and baseline inflammatory markers may be associated with more toxicity among CAR-T patients. Some authors have suggested preemptive treatment with tocilizumab, an IL-6 inhibitor, for patients at higher risk of severe CRS due to higher disease burden, which resulted in a trend for less grade 4 CRS events in a cohort treated with this agent (40). Another study, which investigated fractionated doses of CAR-T cells, showed high CR rates with manageable toxicities in the fractionated dose cohort (41).

Norelli et al. developed a mouse model to recapitulate key features of CRS and found that IL-1 cytokine physiological function abrogation can prevent both CRS and ICANS (42). In their study, the major source of IL-1 and IL-6 during CRS were human monocytes. They were able to prevent CRS by blocking the IL-6 receptor with tocilizumab or by monocyte depletion. However, tocilizumab did not protect mice from delayed lethal neurotoxicity. Instead, the IL-1 receptor antagonist anakinra was able to protect mice from both CRS and neurotoxicity.

A controversial area in the management of CRS/ICANS has been whether the use of tocilizumab and steroids can blunt the efficacy of and responses to CAR-T therapy. A few retrospective analyses (43, 44) have revealed that the use of corticosteroids and tocilizumab do not influence the efficacy and kinetics of CAR-T cell therapy. Few medical centers have adopted the strategy of preemptive use of tocilizumab and/or steroids for mitigation of CD19 CAR-T toxicities, and initial preliminary evidence does not show a detrimental effect on CAR-T therapy efficacy or on responses to this therapy (43). However, in a recent large retrospective analysis of 100 patients with r/r large B-cell lymphoma treated with the CAR-T product axicabtagene ciloleucel, early and prolonged use of high-dose corticosteroids was associated with early progression and death (45). More data are needed to help answer this controversial question.

The CAR-construct can also influence the toxicity profile. CD28 costimulatory domains cause rapid proliferation through the B7 signaling pathway (46), whereas 4-1BB–containing constructs are slower to expand, through activation of the nuclear factor-κB (NF-κB) and tumor necrosis factor receptor–associated factor (TRAF) pathway (47). Third-generation constructs, which combine both CD28 and 4-1BB, have shown better *in-vivo* persistence as well as comparable safety profiles in limited Phase I trials (48, 49); however, further data are needed. The antibody's affinity in CAR-T design can also influence the toxicity profile. A novel second-generation CD19 CAR-T (AUTO1), based on an antibody (Kd ~116 nm) with a faster off-rate but equivalent on-rate compared with conventional FMC63 antibody (Kd ~0.9 nm)-based FDA-approved CD19 CAR-T products, was designed to mimic T-cell kinetics similar to the physiological T-cell activation profile (50). In a clinical trial, AUTO1 CAR showed high efficacy, with a response rate of 83% MRD-negative CR and a favorable toxicity profile despite relatively high tumor burden.

Unified staging scales and management guidelines of CRS and ICANS were recently introduced, thus standardizing patient care (51–54). As the number of patients treated with CAR-T therapy and the number of centers using this therapy have increased, knowledge regarding toxicities relevant to CAR-T therapy and its management has also vastly improved. In future, newer CAR constructs with improved safety profile and greater understanding of clinical management of toxicities will lead to even wider delivery and generalizability of CAR-T therapy.

## Relapse Mechanisms

Relapse after CD19-CAR-T therapy can be broadly categorized into two patterns based on the flow cytometry assessment of CD19 expression on B-ALL: CD19-negative relapses (3, 55, 56) and CD19-positive relapses. Table 2 summarizes the relapse patterns in multiple trials. CD19-positive relapses are usually a function of low potency and poor *in vivo* persistence of manufactured CAR-T cells. Several factors limit the potency and efficacy of CAR-T cells, including the limited long-term persistence (57), the immune-suppressive tumor microenvironment (58), and intrinsic dysfunction associated with T-cell exhaustion (59, 60). Various components of CAR vector constructs such as costimulatory domains (61–63), single-chain variable fragment (scFv) (64), and hinge and transmembrane domains (65, 66) can influence the potency and *in vivo* persistence of CAR-T cells (67). For example, the 4-1BB costimulatory domain ameliorates T-cell exhaustion induced by tonic signaling leading to better *in vivo* persistence (47, 60, 68). Replacement of murine binding domains to the human binding domain in the CAR construct led to lower cytokine levels in the blood and decreased neurotoxicity (65). Increasing the length of the hinge domain in CAR can lead to slow and sustained proliferation without causing neurotoxicity or severe CRS (69). An in-depth review of the mechanistic concept of CAR vector constructs is beyond the scope of this review, and have been detailed elsewhere (67, 70).

**Table 2:** Relapse patterns in B-ALL patients treated with CD19 CAR-T.

**References**	**No. of patients**	**Patients with relapses**	**CD19+ relapses**	**Cd19- relapses**
Maude et al. (1)	30	7	3	4
Jacoby et al. (33)	20	4	3	1
Lee et al. (24)	21	n/a	n/a	2
Park et al. (23)	53	25	9	4
Gardner et al. (27)	43	18	11	7
Turtle et al. (25)	30	9	7	2

Another important aspect of CAR-T cell–based manufacturing that is not well-understood is the influence of age-related immune changes and of patients' previous chemotherapies and other treatment on CAR-T production and efficiency. Guha et al. (71) showed that CAR-T cells from geriatric donors were functionally impaired compared with CAR-T cells from younger donors (71). Compared with geriatric donors, younger donors had higher transduction efficiencies and improved cell expansion with greater cytolytic capabilities. Davila et al. (72) showed that CAR-T cells produced from aged mice showed enhanced cytotoxicity but shorter persistence and a phenotype with less effector memory (73). Also, aging-related T-cell senescence and exhaustion produce significant functional challenges for engineered T-cell therapy (74). This fascinating observation may partly explain why pediatric patients with ALL has better survival outcomes and fewer relapses than the adult/geriatric ALL population.

While most relapses are CD19-positive, some ALL tumors evade CAR-T cell–mediated recognition and clearance by loss of expression of CD19 on the tumor cell surface. Sotillo et al. (75) looked at the genetic/epigenetic mechanisms of CD19-negative relapses by examining tumor samples from patients with CD19-negative disease. In these patients, the authors found deletions in CD19 locus and *de novo* frameshift and missense mutations in exon 2 of CD19. They also discovered lower levels of SRSF3 (a splicing factor whose function is to retain exon 2) in patients with r/r ALL, which allowed exon 2 skipping in tumors, producing a truncated CD19 variant that allowed tumor cells to escape killing by CAR-T cells. According to the authors, the underlying mechanism for relapse in these tumors was the selection of preexisting alternatively spliced variants. Grupp et al. (22) described the phenomenon of “selection by immune pressure” in ALL patients treated with CAR-T cells. They observed the presence of both CD19-negative and positive ALL cells by flow cytometry before CAR-T therapy; later, at the time of relapse, the dominant clone was predominantly CD19-negative, induced by the selective pressure of CD19 CAR-T cells.

Orlando et al. examined specimens by DNA and RNA sequencing from 12 patients who had CD19-negative after CAR-T therapy (76). CD19 mutations were found throughout exons 2–5 in all 12 relapses cases. At least one unique frameshift insertion or deletion was present in each patient. In few cases, missense single nucleotide variants were confirmed as well. In addition, loss of heterozygosity was acquired in 8 of 9 patients at relapse. The allele frequency of mutations measured through DNA sequencing was compared with the percentage of CD19-negative tumor cells by flow cytometry in patients' samples and showed most tumor cells in the relapsed sample contained a CD19 loss-of-function mutation. These findings again confirmed the selective immune pressure of CD19 CAR-T cells. Authors also interrogated mutations in other B cell–specific genes including CD10, CD22, CD20, CD34, CD38, and CD45 and found no mutations associated with relapse. However, contrary to the findings of Sotillo et al. (75), authors found low levels of alternative splicing at extremely low frequencies (0–2.7%) in both initial screening and relapsed samples. This suggests that alternative splicing is incidental to CD19 mutations and may not be involved in tumor evasion of CAR-T cells' immune selection pressure. Future studies exploring the mechanism behind CD19-negative post-CAR-T relapses may help determine whether splicing plays an important role in CD19-negative relapses.

Another mechanism underlying CD19-negative relapses has been ascribed to lineage switch. Jacoby et al. (77), in an ALL mouse model, demonstrated that CAR-T cells create sustained immune pressure against ALL cells with the potential to cause a switch to myeloid lineage markers. Further, they showed that the deletion of Pax5 or Ebf1 recapitulated lineage reprogramming occurring during CD19 CAR immune pressure. Although rare, this lineage switch has also been shown in relapsed human patients (55, 78). Other reported mechanisms of relapse include downregulation of CD22 antigen in loss of response to CD22 CAR-T cells, in a patient who previously lost CD19 expression as well (79), tumor cell–mediated CAR-T trogocytosis (the transfer of the target antigen to the effector T cell) (80) and CAR neutralizing antibody formation (24, 25, 64).

## Allo-HCT After CAR-T Therapy for R/R Adult All

The role of allo-HCT in the remission period after CAR-T therapy is not well-established. CAR-T therapy has immunomodulatory properties, and its associated CRS toxicity with its damaging effect on the endothelium can affect the safety profile of allo-HCT after CAR-T therapy. Moreover, lymphodepletion chemotherapy preceding CAR-T infusion may have an additive effect on allo-HCT–related morbidity and mortality. The specific CAR product used, and treatment population may also be associated with variable transplant outcomes.

Multiple studies have started to establish the safety and efficacy of allo-HCT after CAR-T therapy in r/r ALL patients. In a study conducted at the Fred Hutchinson Cancer Research Center in Seattle, a total of 32 patients (ALL, *n* = 19; NHL/CLL, *n* = 13) underwent allo-HCT after ≥1 CD19-targeted CAR-T infusions with a defined CD4:CD8 ratio (36). The median age at allo-HCT was 46 years (range, 23–74 years). The incidence of grade 3–4 acute graft-vs. host disease (GVHD) and chronic GVHD were 25 and 10%, respectively. One-year treatment-related mortality (TRM) was 21%. The 1-year OS rate was 58%, which is impressive in the r/r ALL setting. An important observation was that longer time from CAR-T therapy to allo-HCT (≥80 vs. <80 days) was associated with a higher risk of death (hazard ratio [HR] 4.01; *P* = 0.03) and a trend toward higher non-relapse mortality (HR 4.4; *p* = 0.19). Overall, the toxicities of allo-HCT in patients who underwent prior CAR-T therapy were not higher than expected in these high-risk patients.

Similarly, in a study from Beijing, China, 52 adult patients with r/r ALL underwent reduced-intensity myeloablative allo-HCT after treatment with either CD19 or CD22 autologous CAR-T cells bearing a 4-1BB costimulatory domain (37). The median time from CAR-T treatment to allo-HSCT was 50 days (range, 34–98 days). The 1-year relapse rate and allo-HCT–related mortality (TRM) were 24.7 and 2.2%, respectively. The incidences of acute and chronic GVHD were comparable to those in previously published studies (81). One-year OS and EFS were impressive at 87.7 and 73.0%, respectively. In this relatively larger cohort, with a quick bridge to allo-HCT after CAR-T therapy, a higher leukemia-free survival was achieved in r/r B-ALL. These studies demonstrate that CAR-T therapy can be used as a quick bridge to allo-HCT in patients with r/r ALL and could potentially augment durable remission rates. In this study, the reduction in dose intensity of the conditioning regimen may have decreased the TRM and increased the OS. The use of reduced intensity conditioning may be a reasonable strategy in these heavily pretreated r/r ALL patients; however, more definitive studies are needed to address this issue.

In pediatric patients with B-ALL, CAR-T therapy has produced more sustained durable responses with lower rates of relapse, compared with rates in adults with B-ALL. For example, in the ELIANA trial (3), the overall remission rate was 81%. Of 75 patients, 45 (60%) had CR, and another 16 (21%) had CRi. However, among the 61 patients who achieved CR or CRi, 22 (36%) experienced relapse. For the whole cohort, the probability of EFS at 12 months was 50%, and median OS was not reached. Eight patients underwent allo-HCT while in remission, and all eight were alive at last follow-up.

In the adult B-ALL population, the durability of response to CAR-T therapy alone has been poor compared with that in the pediatric B-ALL population. Adult r/r B-ALL carries a poor prognosis, with a median survival of less than a year; and less than half of these patients can receive allo-HCT, the only potentially curative modality in these settings (82, 83). Table 3 summarizes the relapse rates and outcomes of r/r ALL after CAR-T therapy in multiple studies and compares the outcomes of adult patients who received post-CAR-T allo-HCT with those of patients who did not receive this therapy. Although CAR-T therapy in adults with r/r ALL has produced a higher CR rate of ~70–90%, more than half of these patients experience relapse within 1 year if CAR-T therapy is not followed by an allo-HSCT (22, 23, 26, 33–37).

**Table 3:** Outcomes in studies with adult patients with B-ALL who received allogeneic allo-HCT after CAR T-cell therapy.

**References**	**Structure of CAR-T**	**Allo-HCT post CAR-T infusion**	
		**Yes**	**No**
Park et al. (23) *n* = 43	CD19-28z	*n* = 17 Relapse, 6/17 (35%); TRM, 6/17 (35%)	*n* = 26 Relapse, 17/26 (65%)
Lee et al. (24) *n* = 51	CD19-28z	*n* = 21 Relapse, 2/21 (9%); LFS not reached (*P* = 0.0006)	*n* = 7 Relapse, 6/7 (86%); LFS, 4.9 mo
Pan et al. (35) *n* = 45	CD19-4-1BB z	*n* = 27 relapse, 2/27 (7%) (*P* = 0.023); TRM, 2/27 (7%); 6-mo LFS, 81.3%	*n* = 18 Relapse, 9/18 (50%)
Pan et al. (34) *n* = 23	CD22-4-1BBz	*n* = 11 relapse, 1/11 (9%); TRM, 2/11 (18%); LFS at 1 year, 71.6%	*n* = 7 Relapse, 4/7 (51%)
Jacoby et al. (33) *n* = 20	CD19-28z	*n* = 14 relapse 2/14 (14%); 1-year EFS, 73%; OS, 90%	*n* = 4 Relapse, 2/4 (50%)
Shalabi et al. (84) *n* = 85 (abstract)	CD19-28z (*n* = 52); CD22-4-1BBz (*n* = 33)	25 went to allo-HCT; 2-year relapse, 13.5%	n/a

*Allo-HCT, allogeneic hematopoietic allo-HCT; EFS, event-free survival; LFS, leukemia-free survival; TRM, treatment-related mortality*.

In one study (23), among 43 ALL patients who had a CR after infusion of CD19 CAR-T cells, 26 were observed with no further therapy and 17 received allo-HCT. The relapse rates in the allo-HCT group (35% [6/17]) were significantly lower those in the no-allo-HCT group (65% [17/26]). However, the significant treatment-related mortality rate of 35% (6/17) in the allo-HCT group, dwarfed the benefits of improvement in relapse. Also, again due to increased TRM in the allo-HCT cohort, in patients who had an MRD-negative CR to CAR-T therapy, no significant difference was observed in EFS and OS between patients who received allo-HCT and those who did not. However, contrary to the above study, most studies have shown significant improvement in survival outcomes in adult patients with r/r ALL who underwent post-CAR-T allo-HCT, as shown in Table 3. For example, in an NCI study (24), 28 of 51 patients achieved MRD-negative CR. The relapse rate (9.5%; 2/21) was significantly less in patients who had undergone allo-HCT after CAR-T therapy than in those who had not (6/7; 85.7%) (*P* = 0.0001). The median leukemia-free survival (LFS) in the allo-HCT group was not reached compared to median LFS of 4.9 months in MRD-negative CR patients who did not proceed to allo-HCT (*P* = 0.0006). In another study, children and young adults (*n* = 85) who were treated with CD19 CAR and CD22 CAR-T cells were pooled for analysis (84). Of 51 patients who attained a CR, 43 were MRD-negative by flow cytometry. Based on competing risk analysis, the 24-month cumulative incidence of post-allo-HCT relapse of all HCT patients was significantly low at 13.5%.

B-cell aplasia (BCA) can be used as a pharmacodynamic measurement of CAR-T persistence (1) since patients with short duration of BCA almost always experience relapse (85). In a phase 1/2 PLAT-02 trial (85), patients with short duration of BCA (<63 days) after CAR-T-infusion had increased risk of relapse. In this study, patients with shorter BCA duration who had attained CR and did not relapse prior to day 63 had significant benefit from consolidative allo-HCT (*P* = 0.007). Of the 15 patients with shorter BCA duration, six did not pursue HCT, and all experienced relapse.

The difference in the CAR-constructs and variability in the patient population makes the cross-study comparison difficult. Overall, all of the above studies highlight the effectiveness of CAR-T therapy in patients with r/r disease and the synergistic role of allo-HCT in the post-CAR-T therapy period. However, prospective trials are needed to define the appropriate role of allo-HCT in the post-CAR-T therapy population. The following is a summary of the important points learned from these trials:

- Adult patients with r/r ALL can achieve unprecedented CR rates with CAR-T therapy and can be transitioned to allo-HCT. Previously, the rate of allo-HCT in r/r has been dismal at 10–30% in some studies (82).
- Despite the use of various targets and costimulatory domains in various CAR constructs, the durability of remission achieved with CAR-T therapy alone in adult patients with r/r B-ALL has been poor, with relapse rates as high as 65–85% in various studies. The 4-1BB costimulatory domain CAR-T shows more durable *in vivo* persistence than does the CD28 costimulatory domain (60).
- Allo-HCT may be associated with more durable remissions and improved overall survival following CAR-T therapies. With increasing depth of remission achieved with CAR-T therapy, we hypothesize that allo-HCT conditioning de-intensification will lead to less TRM and increased OS, especially in patients with second allo-HCT.
- Patients with CNS/leptomeningeal diseases have had excellent responses with CAR-T therapy, despite most of these patients showing evidence of CAR-T cells in cerebrospinal fluid (34, 37). CNS toxicities, including seizures, are higher in patients with evidence of CNS disease, although most of these can be managed with appropriate and timely interventions (34).

## Other Targets for CAR-T Therapy in All

Although the majority of recent clinical trials have focused on CD19 as the target antigen for CAR-T therapy, other targets on B-cell surface markers such as CD20, CD22 could be used to target B-ALL. Besides CD19, the other common target for CAR-T therapy in clinical trials is CD22. In a phase 1 study with CD22 CAR-T cells, where the majority of patients previously failed CD19 CAR-T therapy, the use of CD22 CAR-T cells resulted in a remission in 73% of patients (11/15) (86). Relapses were associated with diminished CD22 density in leukemic cells, which permitted escape from CD22 CAR-T cells.

In another CD22 CAR-T study from China, 34 patients who relapse after CD19 CAR-T therapy achieved 70% CR rates (24/34) (34). Eleven patients (all in CR) went on to receive allo-HCT, and 8 remained in remission at 4.6–13.3 months after allo-HCT with a 1-year leukemia-free survival rate of 71.6% for the whole cohort. Surprisingly, CD22 antigen loss or mutation was not associated with relapse.

To overcome CD19-negative relapses, many research groups have tried to develop dual-target CARs by targeting CD19 and another antigen simultaneously, such as CD22 or CD20 (87–89). Gardner et al. used two lentiviral vectors constructs targeting CD19 and CD22 individually to create a CAR product with three different populations of CAR-T cells (anti-CD19, anti-CD22, and anti-CD19-22) (90). In preliminary results, seven patients were treated, and CR was obtained in 5 (71%), four of whom were MRD-negative. In another phase 1 trial (91), a modified cocktail therapy of CD19 and CD22 CAR was tested in 15 patients with B-ALL. All patients achieved CR or Cri, and 14 were MRD-negative. Among the 15 patients, 11 had an allo-HSCT, and all have remained in remission at the time of manuscript submission. In another phase 1 study (91, 92) of a bicistronic CAR-T targeting CD19 and CD22 in r/r B-ALL, seven evaluable patients all achieved a remission. At a median follow-up at 8 months, three relapses had occurred, including one with CD19-negative/CD22-low expression. A recently published study of clinical trial using CD19/CD22 dual CAR-T cells with a 4-1BB co-stimulatory domain showed all seven of the patients in the second dose cohort achieving CR, with six of them being MRD negative CR (93).

Other trials targeting dual antigens are currently under way (30), including preclinical data regarding a dual CD19 and CD123 targeting CAR (94). CAR-T therapy targeting 3 targets–CD19, CD20, and CD22, are also under development for ALL (95). A chondroitin sulfate proteoglycan 4 (CSPG4) membrane surface receptor has been found on mixed lineage leukemia (MLL) rearranged B-ALL cells. A CSPG4-specific CAR is an active area of investigation for MLL rearranged B-ALL (96).

## Future Directions and Conclusions

Over the past 50 years, we have seen several breakthroughs in the treatment of B-ALL, especially in childhood B-ALL; however, CAR-T therapy represents a significant innovation and a major milestone in the treatment of both pediatric and adult B-ALL. Relapses after CAR-T therapy and poor persistence of CAR-T cells *in vivo* have emerged as major obstacle to widespread success in B-ALL patients who undergo this therapy. Novel strategies are being implemented to not only increase the potency and persistence of CAR-T cells but also decrease the toxicities to make the use of CAR-T therapy safer.

New cancer-associated antigens are being explored as potential targets for CAR-T cells. Also, multi-targeted CARS are now being tested in early-phase studies, in an effort to reduce antigen loss as a resistance mechanism (92, 95, 97). Various components of CAR constructs are being enhanced to maximize their potential and synergize with the tumor microenvironment. For example, the higher affinity of the T-cell receptor may at times actually impair the selectivity of the cells and reduce overall CD8 T-cell function (98, 99). One study showed that a lower-affinity ScFv CAR construct showed better proliferation than did higher-affinity ScFv CAR constructs and produced MRD-negative remission in 12 of 14 patients treated, five of whom had continuous remissions at a median of 14 months of follow-up (64). CAR constructs with cytokine secretion and immune modulation, termed fourth-generation or armored CARs, are being developed to further augment CAR-T activity (100, 101). Some of these novel CAR constructs use paracrine signaling, whereas others activate immune cells or counteract immune rejection through PD-1 blockade and other immunoregulatory mechanisms (100, 102, 103). Checkpoint inhibitors have also been combined with CAR-T cells to improve efficacy. In one study, ALL patients who lost B-cell aplasia after CAR-T therapy were treated with checkpoint inhibitors, and three of six patients had reacquired B-cell aplasia after the treatment (104).

Multiple other strategies to enhance CAR-T cell expansion and persistence are being devised including overexpression of certain genes such as c-jun (59), CRISPR knockouts, TET2 gene disruption (105), enzyme overexpression to metabolically engineer against the tumor microenvironment (106), and expression of erythropoietin receptor in CAR constructs with the ability to expand *in-vivo* with erythropoietin. In another example of expanding and enhancing the persistence of CAR-T cells, patient-derived antigen-presenting cells were transduced with a lentiviral vector coding a truncated CD19 (CD19t) (107) and were infused into patients at high risk of short CAR-T cell persistence, such as low antigen tumor burden, rapid CAR-T contraction, or an early loss of B cell aplasia. All 11 patients had an increase in CD19 CAR-T cells, with 5 of 10 having ongoing B-cell aplasia with a median follow-up of 8.8 months.

Systemic toxicities from CAR-T therapy is another major hurdle in the developing CAR-T field, and multiple avenues are being explored to make CAR safer. Ying et al. devised a new CD19 CAR construct with a longer CD8α hinge length (86 amino acids) and found that CAR-T cells transduced with this construct produced lower levels of cytokines and proliferated at a slower pace than did prototypical CD19 CAR-T cells (69). In a phase 1 trial, 6 of 11 patients achieved CR and notably, no neurological toxicity and no severe CRS (greater than grade 1) occurred in any patient. Similarly, a clinical trial using a CAR containing a fully human scFv targeting CD19 demonstrated lower neurotoxicity rates than did a cohort using a murine scFv, due to lower cytokine secretion by the human scFv–containing cells (65). The investigators at the University of Pennsylvania have tried fractionated infusions of CAR-T cells split over 3 days, which allowed for day 2 and 3 doses to be held for early CRS, and found that high-dose fractionated dosing of CD19 CAR with patient specific dose modification optimizes safety without compromising efficacy (41).

Another hurdle for CAR-T therapy that limits its broad applicability is the long manufacturing process, which not only is costly but also leads to a more exhausted CAR-T phenotype in the final product. A new “FasT” platform, which uses electroporation to transduce the CAR gene and has shortened the CAR-T cell manufacturing process by more than 24 h, has shown superior expansion capability and younger/less exhausted phenotypes in a phase I clinical trial (108). Initial clinical reports have been encouraging: CR was achieved in all 10 treated patients, nine of whom were MRD-negative.

Other ways to mitigate this obstacle is to develop allogeneic “off the shelf” therapies (109); however, allogeneic cells bear the risk of immune rejection by host T cells, as well as allo-reactivation of the CAR-T cells via the TCR receptor against host tissues, causing GVHD (110). Many trials are currently enrolling “off the shelf” products, including a few trials with gene-edited deletion of the surface TRAC molecule to prevent GVHD (111); however, preliminary results reveal responses to be short-lived. How successful these “off the shelf” therapies will be in the future is still an open question. Despite the multiple limitations described in this paper, the CAR-T therapy field has continued to progress with significant innovation and holds great promise to revolutionize our approach to cancer treatment.

## Author Contributions

UG and NS contributed with drafting the paper, and writing the manuscript. PK, KM, and ES participated in the critical revision of the article. All authors reviewed and approved the manuscript.

## Conflict of Interest

The authors declare that the research was conducted in the absence of any commercial or financial relationships that could be construed as a potential conflict of interest.
